# Genetic Prediction of Future Type 2 Diabetes

**DOI:** 10.1371/journal.pmed.0020345

**Published:** 2005-11-01

**Authors:** Valeriya Lyssenko, Peter Almgren, Dragi Anevski, Marju Orho-Melander, Marketa Sjögren, Carola Saloranta, Tiinamaija Tuomi, Leif Groop, the Botnia Study Group

**Affiliations:** **1**Department of Clinical Sciences, Diabetes and Endocrinology, Lund University, University Hospital Malmö, Malmö, Sweden,; **2**School of Mathematical Sciences, Chalmers University of Technology, Gothenburg, Sweden,; **3**Department of Medicine, Division of Diabetology, Helsinki University Hospital, Helsinki, Finland,; **4**Folkhälsan Research Center, Institute of Genetics, Helsinki, Finland; Peninsular Medical School, ExeterUnited Kingdom

## Abstract

**Background:**

Type 2 diabetes (T2D) is a multifactorial disease in which environmental triggers interact with genetic variants in the predisposition to the disease. A number of common variants have been associated with T2D but our knowledge of their ability to predict T2D prospectively is limited.

**Methods and Findings:**

By using a Cox proportional hazard model, common variants in the *PPARG* (P12A), *CAPN10* (SNP43 and 44)*, KCNJ11* (E23K)*, UCP2* (−866G>A), and *IRS1* (G972R) genes were studied for their ability to predict T2D in 2,293 individuals participating in the Botnia study in Finland. After a median follow-up of 6 y, 132 (6%) persons developed T2D. The hazard ratio for risk of developing T2D was 1.7 (95% confidence interval [CI] 1.1–2.7) for the *PPARG* PP genotype, 1.5 (95% CI 1.0–2.2) for the *CAPN10* SNP44 TT genotype, and 2.6 (95% CI 1.5–4.5) for the combination of *PPARG* and *CAPN10* risk genotypes. In individuals with fasting plasma glucose ≥ 5.6 mmol/l and body mass index ≥ 30 kg/m^2^, the hazard ratio increased to 21.2 (95% CI 8.7–51.4) for the combination of the *PPARG* PP and *CAPN10* SNP43/44 GG/TT genotypes as compared to those with the low-risk genotypes with normal fasting plasma glucose and body mass index < 30 kg/m^2^.

**Conclusion:**

We demonstrate in a large prospective study that variants in the *PPARG* and *CAPN10* genes predict future T2D. Genetic testing might become a future approach to identify individuals at risk of developing T2D.

## Abstract 2

In a large prospective study, Lyssenko and colleagues show that variants in the PPARG and CAPN10 genes can help predict whether a person will develop Type 2 diabetes.

## Introduction

Type 2 diabetes (T2D) is a multifactorial disease in which environmental triggers interact with genetic variants in the predisposition to the disease [1]. T2D is characterized by impaired insulin secretion and insulin action in target tissues such as muscle and liver [2]. Many patients with a genetic predisposition to T2D also have a predisposition to weight gain, and obesity is a strong risk factor for T2D [3].

Although several candidate genes have been associated with T2D [4–7], many findings have been difficult to replicate. The list of genes with support in extensive meta-analyses is relatively short, including genes encoding for PPARG, calpain 10, Kir 6.2, and insulin receptor substrate 1 (IRS1) [8]. The *PPARG* P12A polymorphism is associated with enhanced insulin sensitivity and protects against T2D [4,9–11]. Although the individual risk reduction for carriers of the rare A allele is only 15%, the population attributable risk of the common allele is about 25%. Two intronic single nucleotide polymorphisms (SNPs) (43 and 44) in the gene encoding for the cystein protease calpain 10 *(CAPN10)* confer increased susceptibility to insulin resistance and T2D [12–15]. The ATP-sensitive potassium channel Kir 6.2 *(KCNJ11)* forms together with the sulfonylurea receptor SUR1 *(ABCC8),* an octamer protein that regulates transmembrane potential and thereby glucose-stimulated insulin secretion in pancreatic β-cells. A E23K polymorphism in *KCNJ11* has been associated with T2D [16–18]. Carriers of a G972R polymorphism in the IRS1 gene *(IRS1)* have been shown to have reduced insulin content in pancreatic islets [19]. Although the meta-analyses suggested a role for the G972R polymorphism in T2D [8,20,21], two recent large case-control studies failed to replicate this association [22,23].

In addition to the genes listed above, we considered it worthwhile to also include the uncoupling protein 2 gene *(UCP2)* in the analysis because some studies have associated a polymorphism in the promoter of the gene (*UCP2* −866G>A) with increased risk of T2D and impaired insulin secretion [24–27], whereas other studies have reported reduced risk of T2D [28]. Increased expression of UCP2 in pancreatic islets is associated with increased uncoupling and thereby decreased ATP production required for insulin secretion [29].

In this study, we tested variants in a number of candidate genes for T2D for their ability to predict diabetes in 2,293 individuals without diabetes participating in the Botnia prospective study in western Finland.

## Methods

### Study Participants

The Botnia study is a family-based study aiming to identify genes increasing susceptibility to T2D [30,31]. Details of the study cohort and sampling strategy have been presented earlier [31]. In brief, individuals with T2D from the area of five health-care centers in western Finland were invited to participate, together with their family members [31]. An oral glucose tolerance test (OGTT) was performed for all participants aged 18–70 y who had fasting plasma glucose concentration (FPG) lower than 11 mmol/l. Participants without diabetes, either family members of T2D patients or control participants (spouses without first or second degree family history of diabetes), between 18–70 y were offered prospective visits every 2–3 y. During the study period (which started in 1990 and was closed for this analysis in 2002), 1,869 relatives of T2D patients from 577 extended pedigrees (approximately three persons per pedigree) and 424 controls without family history of diabetes participated in at least OGTTs with a median follow-up of 6 y (range 2–12 y). Of the participants in both these groups, 1,569 had normal glucose tolerance and 724 had impaired fasting glucose and/or impaired glucose tolerance at baseline. Carriers of mutations causing maturity onset diabetes of the young (*n =* 20) were excluded from the present study. Glucose tolerance was defined according to the current World Health Organization criteria [32]. All participants gave informed consent, and the local ethics committee approved the study.

### Anthropometric Measurements and Assays

The participants' weight, height, waist and hip circumference, and blood pressure were measured as previously reported [30,31]. Body mass index (BMI) was calculated as weight (in kilograms) divided by height (in meters) squared. All participants participated in a 75-g OGTT after a 12-h overnight fast. Fasting blood samples were drawn for the measurement of high density lipoprotein cholesterol, triglyceride, and free fatty acid concentrations, and at −10, 0, 30, 60, and 120 min for the measurement of plasma glucose and serum insulin. Insulin resistance was estimated as homeostasis model assessment index (HOMA_IR_) using a computer-based model [33] and β-cell function as the ratio of incremental insulin to glucose responses during the first 30 min of the OGTT (ΔI/ΔG = ΔI_30 min fasting_/ΔG_30 min fasting_); this index is also called the insulinogenic index. The disposition index was used to adjust insulin secretion for the degree of insulin resistance (insulinogenic index/HOMA_IR_).

### Genotyping

Genotyping of SNPs was performed with a polymerase chain reaction–restriction fragment length polymorphism (PCR-RFLP) method and agarose gel electrophoresis for *IRS1* G972R, or using the Multiplex SNaPshot kit (Applied Biosystems, Stockholm, Sweden) for single base pair extension on ABI 3100 (Applied Biosystems) for *CAPN10* SNP43 and 44, or with an allelic discrimination assay-by-design method on ABI 7900 (Applied Biosystems) for *KCNJ11* E23K, *IRS1* G972R, and *UCP2* −866G>A (Table S1). By randomly regenotyping 10%–20% of the samples, we achieved concordance rates of 99% for *PPARG* P12A, *CAPN10* SNP44, *KCNJ11* E23K, *IRS1* G972R, and *UCP2* −866G>A, and 98% for *CAPN10* SNP43. All genotypes were in Hardy–Weinberg equilibrium except *CAPN10* SNP44, which showed a moderate deviation from equilibrium (*p =* 0.035). Genotyping errors are an unlikely explanation for this deviation from equilibrium, because in genotyping 1,880 samples of *CAPN10* SNP44 using two different methods (allelic discrimination and single base extension) the concordance rate was 99%.

### Statistical Analyses

Variables are presented as median (interquartile range). A χ^2^ test was used for comparison of group frequencies. Survival analysis was used to estimate the effect of genetic variants (risk and non-risk genotypes defined from previous studies) on the risk of developing T2D and shown by Kaplan–Meier survival curves as the distribution of age at onset (the proportion of individuals developing T2D at certain age). The risk of developing T2D was expressed as a hazard function (the negative slope divided by the survival curve) using an age-adjusted Cox proportional hazard regression model [34]. The hazard function is the (conditional) probability for the development of diabetes during a time interval divided by the length of that time interval, for an individual that is diabetes-free at the start of the time interval. The relative effect is presented as the ratio between the hazard functions (hazard ratio [HR]) of the two groups. HRs quantify the effect size of both discrete variables (carriers versus non-carriers) and continuous variables (used in the interaction analysis below where HR measures the effect of an increase in one unit of the continuous variable). All survival analyses were stratified for gender and adjusted for family history of diabetes and BMI (when appropriate). The information that an individual did not or did belong to a nuclear family with at least one other affected member was coded as zero or one, respectively, and used as a covariate in the Cox regression analyses. All survival analyses were performed with a robust variance estimate to adjust for within family dependence extended to the large pedigrees. In using a robust variance estimate we treated each pedigree (instead of each individual) as an independent entity for calculating the variance of the estimates.

Expected risk genotypes were defined according to earlier reports (PP genotype of *PPARG,* GG genotype of *CAPN10* SNP43, TT genotype of *CAPN10* SNP44, EK/KK genotypes of *KCNJ11,* GR/RR genotypes of *IRS1,* GG genotype of *UCP2*). However, the risk TT genotype of *CAPN10* SNP44 was in opposite direction compared to other studies [13,14] and selected based upon a previous report from the Botnia study [15] showing that the combination of the TT genotype of SNP44 and the GG genotype of SNP43 in *CAPN10* was significantly more frequent in patients with T2D than in control individuals. Therefore, in the present study we refer to the TT genotype of *CAPN10* SNP44 as an at-risk genotype. Individuals with missing data were excluded from the analyses.

Analyses of interaction between effect of phenotype *(P)* defined as insulin secretion (disposition index) and insulin action (HOMA_IR_) and genotype *(G)* (1 = risk and 0 = non-risk) on age at onset of T2D were performed using the following Cox proportional hazards model: h(*t*) = h_0_(*t*)exp(β_1_ *P* + β_2_ *G* + β_3_ P*G), in which h(*t*) is the hazard function and h_0_(*t*) is the baseline hazard function, with β_1_ and β_2_ measuring the univariate effects and β_3_ measuring the interaction. If there is an interaction (β_3_ ≠ 0), the HR for carriers and non-carriers of the risk genotype will not be the same. Thus, in different genotype carriers the HR of T2D associated with *x* units increase/decrease in the phenotypic value *(P* + *x)* is HR = exp(β_1_ + β_3_)*x for the risk genotype carriers and HR = exp(β_1_*x) for the non-risk genotype carriers. A logistic regression analysis was applied to explore the relationship between FPG and BMI, with genetic factor (defined as 1 = risk and 0 = non-risk) as dependent variable and FPG, BMI, and an interaction term as covariates. All statistical analyses were performed using Number Crunching Statistical Systems version 2004 (NCSS, Kaysville, Utah, United States), R (www.r-project.org, and Stata (StataCorp, College Station, Texas, United States). Two-sided *p*-values of less than 0.05 were considered statistically significant.

## Results

In total, 2,293 persons (1,051 men and 1,242 women) were included in the study (Table 1). Of them, 1,078 (47%) had non-normal FPG (≥ 5.6 mmol/l), 280 (12.3%) had BMI ≥ 30 kg/m^2^, and 160 (7%) had both elevated FPG and BMI ≥ 30 kg/m^2^. Of the 2,293 persons included, 132 (6%) (67 men and 65 women; 40 with normal and 92 with abnormal glucose tolerance) developed diabetes during the follow-up period of 6 y (converters).

**Table 1:**
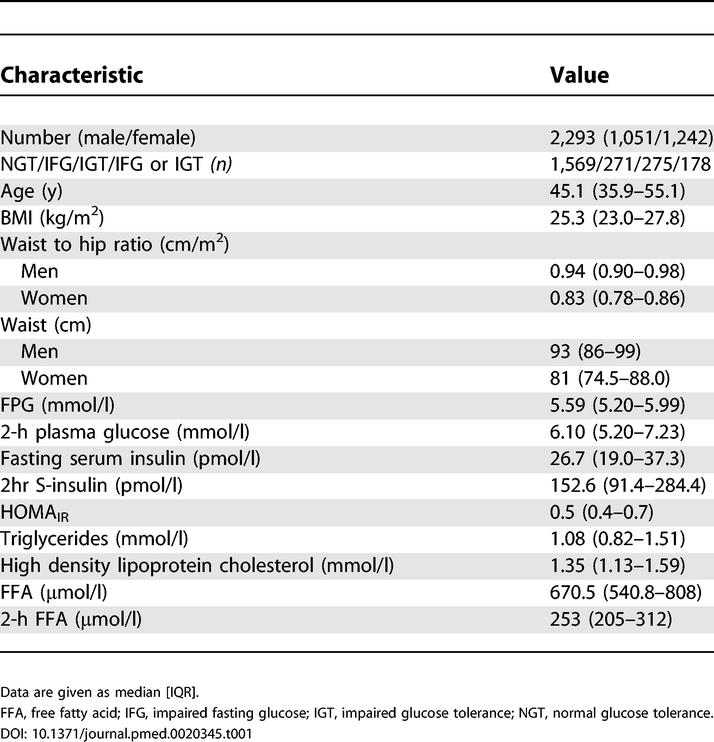
Clinical Characteristics of the Participants at Baseline

### *PPARG*

**Figure 1:**
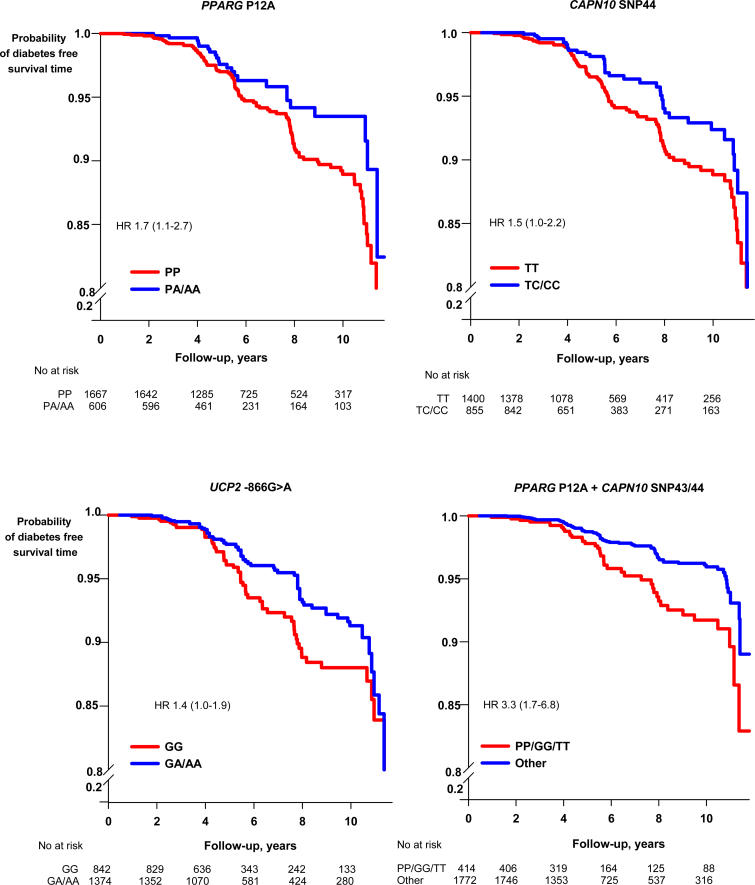
Unadjusted Kaplan–Meier Diabetes-Free Survival Probability Curves Curves for different carriers of *PPARG* P12A (PP versus PA/AA), *CAPN10* SNP44 (TT versus TC/CC), *UCP2* −866 G/A (GG versus GA/AA), and the combination of *PPARG* and *CAPN10* SNP43/44 (PP/GG/TT versus other). *y-*Axis shows probability of diabetes-free survival time. *x*-Axis shows follow-up time in years. The HR of developing T2D in different genotype carriers obtained from Cox proportional hazards regression stratified on sex and adjusted for age, BMI, and family history of diabetes with robust variance estimate is shown (see also Table 3).

**Figure 2:**
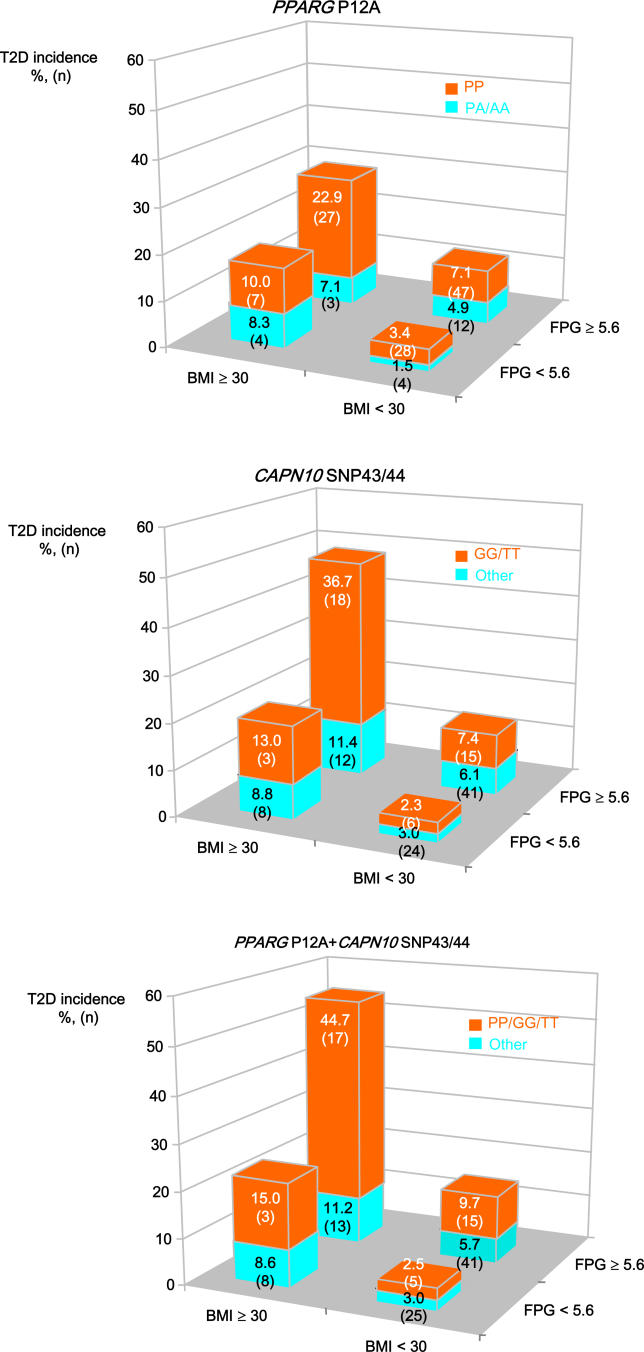
The Effects of Risk Genotypes of the *PPARG* P12A Polymorphism (PP), the Combination of *CAPN10* SNP43/44 (GG/TT), and the Combination of *PPARG* and *CAPN10* SNP43/44 (PP/GG/TT) Together with FPG and BMI for the Risk of Developing T2D *y*-Axis denotes incident diabetes estimated as the proportion (percent) of participants who developed diabetes during the follow-up period in the groups with each risk factor defined as risk genotype, elevated FPG (≥5.6 mmol/l), and high BMI (≥30 kg/m^2^). The absolute number of individuals who developed diabetes in the groups with each risk factor is given within the bars (in parentheses) and in Table S2. The incidence of T2D was significantly increased in carriers of the risk PP genotype, GG/TT genotypes, and PP/GG/TT genotypes with elevated FPG and high BMI as compared with individuals carrying low risk genotypes without risk factors (χ^2^ test, *p <* 0.001).

**Figure 3:**
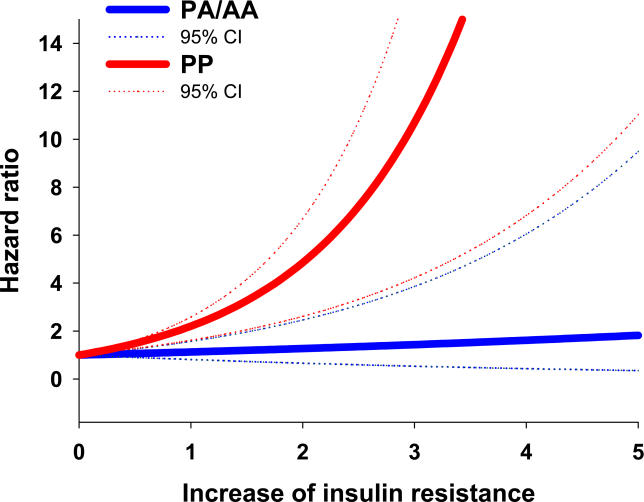
The Effect of Insulin Resistance Together with the Risk Genotype of the *PPARG* P12A Polymorphism on Risk of Developing T2D *y*-Axis denotes HR and its 95% CI. *x*-Axis denotes increase in insulin resistance estimated as HOMA_IR_.

The allele and genotype frequencies of the *PPARG* P12A polymorphism were similar to those previously reported in Caucasians [4], with 73.3% of participants carrying the risk PP genotype (Table 2). Of all individuals who developed T2D, 109 (82.6%) had the PP genotype, which also significantly increased the risk of subsequent T2D (HR 1.7, *p =* 0.016) (Figure 1; Table 3). Because we have previously shown that a family history of diabetes, non-normal FPG (≥ 5.6 mmol/l), and BMI ≥ 30kg/m^2^ identify individuals at high risk of T2D [31], we now tested whether the *PPARG* risk genotype could replace family history in this prediction. In fact, the incidence of T2D was increased in carriers of the PP genotype with elevated FPG and high BMI as compared with the PA/AA genotype carriers without any other risk factors (22.9% versus 1.5%, *p <* 0.001) (Figure 2). This corresponded to a HR of 13.5 (95% confidence interval [CI] 4.5–40.7, *p <* 0.001) estimated by the Cox model (Table 3). The *PPARG* genotype also influenced the relationship between BMI and FPG; there was a stronger correlation between BMI and FPG in carriers of the *PPARG* PP as compared to the PA/AA genotypes (0.23 versus 0.15, *p =* 0.041), suggesting a steeper increase in FPG for any increase in BMI in carriers of the risk genotype. Furthermore, we observed a significant interaction between the *PPARG* P12A polymorphism and HOMA_IR_ (*p =* 0.004), indicating that with increasing insulin resistance [31] carriers of the PP genotype had a greater risk of developing T2D than carriers of the PA/AA genotypes (Figure 3).

**Table 2:**
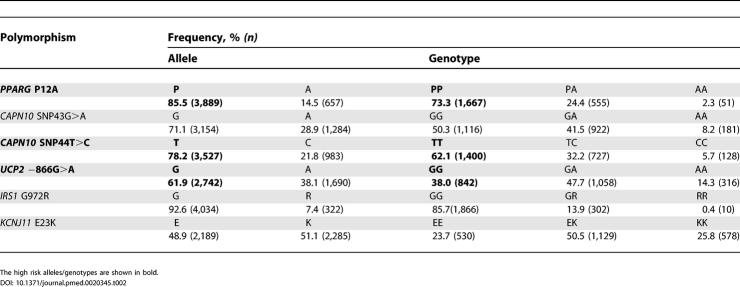
Allele and Genotype Frequencies of the Studied Polymorphisms

**Table 3:**
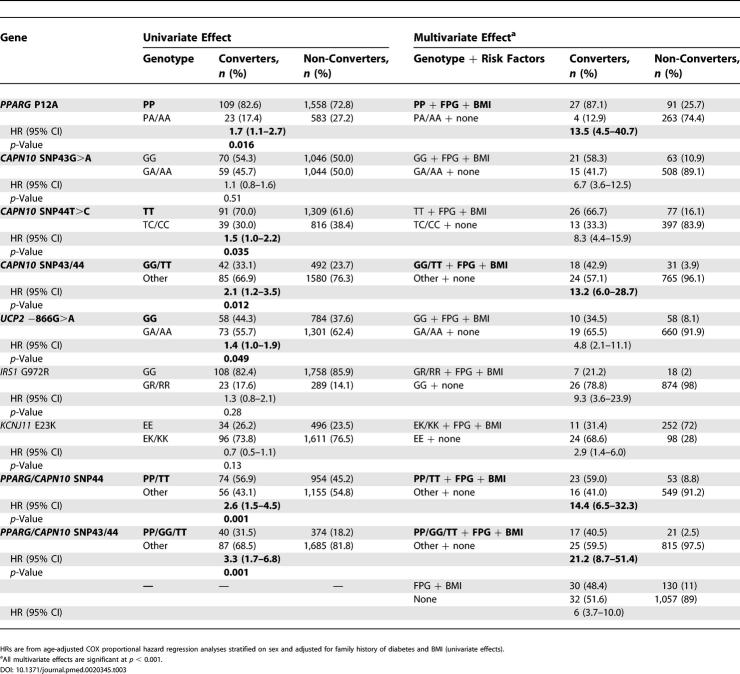
Risk of Developing T2D in Different Genotype Carriers of the Studied Polymorphisms

The G allele of SNP43 and the C allele of SNP44 were in strong linkage disequilibrium (*D*′ = 0.99, *p <* 0.001). Fifty percent of the participants had the risk genotype (GG) of SNP43 and 62.1% had the risk genotype (TT) of SNP44. A total of 534 (24%) individuals carried both the GG (SNP43) and the TT (SNP44) genotypes. Seventy (54.3%) of the converters had the GG (SNP43) genotype, while 91 (70.0%) had the TT (SNP44) genotype. While SNP43 had no effect on its own, the SNP44 TT genotype was associated with a moderately increased risk of T2D (HR 1.5, *p =* 0.035) (see Figure 1; Table 3). The incidence of T2D was highest in individuals carrying both SNP43 (GG) and SNP44 (TT) genotypes and other risk factors, particularly those with high BMI, as compared with individuals with low risk genotypes without any other risk factors (36.7% versus 3.0%, *p <* 0.001) (see Figure 2). In the age-adjusted Cox analysis, the corresponding HR was 13.2 (95% CI 6.0–28.7, *p <* 0.001) (Table 3). Also, there was a significant interaction between the GG genotype of SNP43 and HOMA_IR_, and between the combination of both risk genotypes (GG/TT) and HOMA_IR_ (*p =* 0.037 and *p =* 0.028, respectively), showing that the risk conferred by worsening of insulin sensitivity [31] increased more in carriers of these genotypes than in carriers of the low risk genotypes.

### *UCP2*

Fifty-eight (44.3%) of the converters were homozygous for the risk genotype (GG) of the *UCP2* −866G>A variant. The GG genotype was associated with a modestly increased risk of T2D (HR 1.4, *p =* 0.049) (see Figure 1; Table 3). This risk was not influenced by BMI and FPG at baseline. However, the GG genotype was also associated with increased risk of developing T2D in patients with earlier onset of diabetes (HR 2.0, 95% CI 1.2–3.3, *p =* 0.0057) (Table 4). Furthermore, the GG genotype was also more frequent among patients with earlier than late onset of T2D (60.3% versus 39.7%, *p =* 0.042; χ^2^ test and odds ratio 2.5, 95% CI 1.2–5.3, *p =* 0.016; logistic regression analyses adjusted for gender, BMI, and family history of diabetes). None of the other tested genotypes predicted significantly earlier onset of T2D.

**Table 4:**
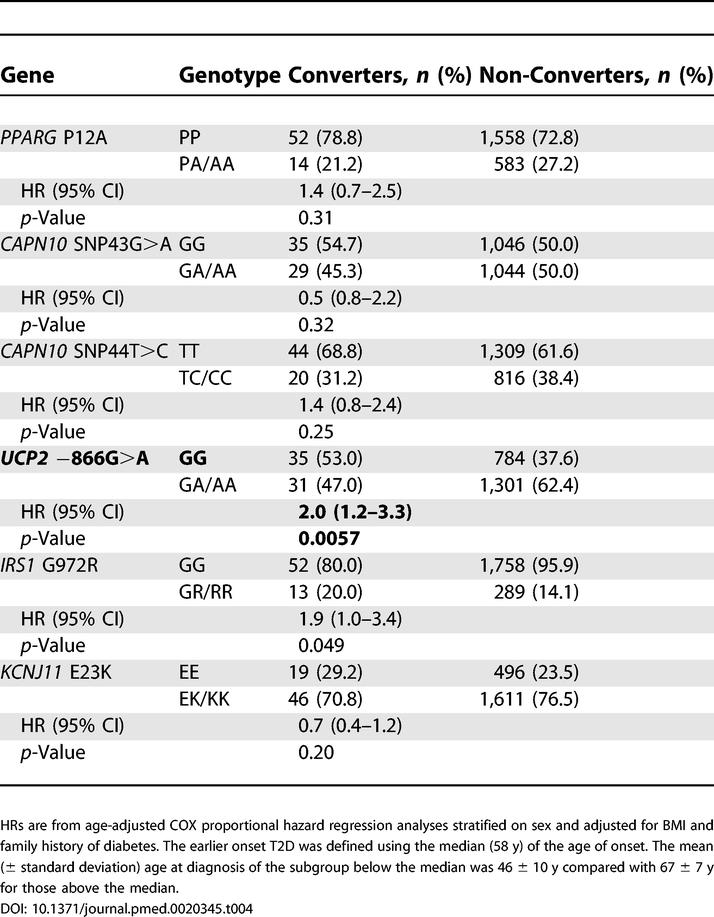
Risk of Developing Earlier Onset T2D in Different Genotype Carriers of the Studied Polymorphisms

### *IRS1*

Twenty-three (17.6%) converters carried the RR/RG genotypes of the *IRS1* G972R polymorphism. Whereas the R allele had no independent effect on T2D risk, it increased the risk of T2D in a dominant fashion (RR or RG versus GG) in participants with elevated FPG and BMI ≥ 30 kg/m^2^ to 9.3 (95% CI 3.6–23.9, *p <* 0.001) (see Table 3).

### *KCNJ11*

Ninety-six converters (73.8%) had the risk EK/KK genotypes of the *KCNJ11* E23K polymorphism, but these genotypes did not increase risk of future T2D, neither alone nor in combination with elevated FPG or high BMI. In line with previous findings of an effect of this variant on insulin secretion [16–18], there was a significant interaction between the *KCNJ11* E23K polymorphism and the disposition index (*p <* 0.001), suggesting that the risk of T2D associated with a low disposition index [31] is increased by the EK/KK genotypes.

### Combined Genetic Effects

In total, 1,028 (45.9%) individuals carried risk genotypes in both *PPARG* (PP) and *CAPN10* SNP44 (TT), whereas 397 (18.2%) individuals had three risk genotypes: *PPARG* (PP), *CAPN10* (TT), and *UCP2* (GG). The effect of both the *PPARG* PP and *CAPN10* SNP44 TT genotypes on the risk of subsequent T2D when present in the same individual was greater (HR 2.6, 95% CI 1.5–4.5) than the individual risks (Table 3). The effect was even stronger when the combination of at-risk GG and TT genotypes of both SNP43 and 44 of the *CAPN10* gene (HR 3.3, 95% CI 1.7–6.8) (Table 3; see Figure 1) was included in the analysis. Again, the incidence of T2D in participants with the combination of SNP43 (GG) and SNP44 (TT) in *CAPN10,* the PP genotype in the *PPARG* gene, elevated FPG, and high BMI was markedly higher than in those with low risk genotypes and no other risk factors (44.7% versus 3.0%, *p <* 0.001) (see Figure 2), with a HR of 21.2 (95% CI 8.7–51.4, *p <* 0.001) (Table 3). Also, these combinations influenced the correlation between BMI and FPG, yielding a steeper increase in FPG for any increase in BMI in carriers of the risk genotype combinations than in carriers of the non-risk genotypes (0.28 versus 0.19, *p =* 0.041).

## Discussion

The key finding of the present study was that variants in the *PPARG* and *CAPN10* genes increased the future risk for T2D, particularly in individuals with other risk factors. A question often raised about genetic association studies of polygenic diseases is whether the information can be used to predict the disease, since the risk conferred by the variant is usually rather modest (odds ratio < 1.2–1.3) [6]. In T2D association studies, cases are usually ascertained through diabetes clinics and thereby possibly enriched by carriers of more severe genetic variants. It was therefore encouraging to see that polymorphisms in some genes previously shown to be associated with T2D in case-control studies (particularly P12A in *PPARG* and SNP44 in *CAPN10*) [4,15] could predict T2D in high risk individuals from families with T2D.

The relative risk (λ_s_) of developing T2D for members of the Botnia families is about three. However, this risk is greatest in obese (BMI ≥ 30 kg/m^2^) individuals with FPG above normal (≥5.6 mmol/l) and a family history of T2D [31]. Replacing the family history with the *PPARG* and *CAPN10* variants, and particularly with their combination, gave almost the same strong prediction of subsequent T2D. These genotypes also influenced the relationship between BMI and FPG, i.e., in carriers of the risk genotypes there was a steeper increase in FPG for any given increase in BMI.

Several papers have examined the effect of single gene variants on the risk of conversion to T2D in interventional trials like the Finnish Diabetes Prevention Study [9,17] and the STOP-NIDDM trial [11]. However, it is important to know the effect of these genetic variants on risk of future T2D in an observational study before conclusions can be drawn on their putative additive or synergistic effects, together with the effects of specific factors such life style changes [9,17] or acarbose use [11]. Many of these studies have provided conflicting results in different subgroups; this is a natural corollary of their design, which breaks the initial cohorts down in relatively small subgroups with limited power. Although the present study also has limited power, it is to our knowledge the largest of its kind, and it also provides information on key T2D variants in the same paper.

### *PPARG*

The P12A polymorphism in *PPARG* is to date the best replicated genetic variant for T2D, with a cumulative odds ratio from published studies of about 1.25 and overall *p <* 0.001 [4,8]. The P12A variant is located in an extra exon B in the 5′-end of the adipose-specific *PPARG*2 isoform and shows reduced transcription of target genes. The A allele has been associated with increased insulin sensitivity [35], particularly, enhanced suppression of lipolysis [36]. In support of this, there was a significant interaction between the P12A polymorphism and HOMA_IR_ (which measures insulin resistance). The Nurse's Health Study also reported reduced risk for developing diabetes in carriers of the A allele [10]. Most recently, the STOP-NIDDM trial also reported that the PP genotype predicted conversion to diabetes in women in the acarbose intervention group [11]. These combined data, however, contrast with findings in the Finnish Diabetes Prevention Study, which reported an increased risk of developing diabetes in carriers of the A allele compared to individuals with the P12P genotype [9]. This effect was restricted to the control group, whereas the few A12A carriers in the intervention group lost more weight than the P12P carriers. As enhanced insulin sensitivity is a risk factor for weight gain, the A allele has also been associated with more rapid weight regain after weight reduction [37]. It is therefore possible that the protective effect of the A allele is attenuated in very obese individuals. Differences in BMI cannot fully explain the different results, since the risk conferred by the P12P genotype was maintained after adjusting for BMI in the present study. The effect of the P12A variant on BMI and lipolysis is also dependent upon intake of saturated fat [38]. Taken together, the data suggest a complex interaction between the P12A polymorphism in the *PPARG* gene and diet, body weight, and insulin sensitivity in predicting risk of future T2D.

Notably, the HR in the present prospective study was higher than the odds ratios in previous case-control studies [4]. The same also applies to the odds ratios obtained in the intervention studies discussed [10,11]. Although there could be several possible explanations for this discrepancy, a likely explanation is the accuracy by which the cases (converters) and controls (non-converters) were defined in the prospective study as compared with a case-control association study. In the prospective study all individuals underwent repeated OGTTs to define glucose tolerance status, while in case-control studies the definition of normal glucose tolerance is often based upon one OGTT. Therefore, we assume that we have a certain proportion of controls misclassified. We simulated (1,000 times) this situation by introducing 5%, 10%, and 20% misclassification of non-converters regarding the HR of 1.72 for the P12A polymorphism in the *PPARG* gene to predict future diabetes. A 5% misclassification would result in a decrease in HR to 1.31 (minimum 0.85; maximum 1.97), 10% to 1.18 (minimum 0.84; maximum 1.68), and 20% to 1.08 (minimum 0.82; maximum 1.38). Of course, there also could be other factors contributing, such as change in diabetes prevalence (which almost doubled) between the time when the cases in the case-control study and the converters in the prospective study were diagnosed. Finally, our study was carried out in a high risk population of first degree relatives of patients with T2D.

### *CAPN10*

The discovery that intronic SNPs in the *CAPN10* gene explained the linkage to Chromosome 2q in Mexican-Americans represented the first successful positional cloning of a T2D gene [12]. It also raised a number of questions, e.g., how could these intronic SNPs in a gene encoding a cystein protease confer increased risk of T2D? Several recent meta-analyses have demonstrated a consistent but modest risk of SNP43 and 44 (odds ratio 1.15–1.20) for the association with T2D [13,14]. In opposite direction to other studies [13,14], in the Botnia study the TT genotype of SNP44 has been associated with increased risk of T2D [15]. However, a stronger association was seen for the combination of both the GG genotype of SNP43 and the TT genotype of SNP44 [15]. Carriers of the GG genotype of SNP43 have decreased *CAPN10* mRNA levels in skeletal muscle, which correlates with more severe insulin resistance [39]. Our data of an interaction between HOMA_IR_ and the *CAPN10* SNP43 supports this notion. The question arises whether the combined effect of variants in the *PPPARG* and *CAPN10* genes on risk for future T2D can be solely explained by the variants' effect on insulin sensitivity. A combined effect of the two genes on both insulin secretion and action would be more plausible. However, the knowledge of molecular mechanisms by which calpain 10 would increase susceptibility to T2D is limited.

### *UCP2*

Impaired insulin secretion has been shown to predominate over insulin resistance in individuals with early onset T2D [40]. In line with this view, the *UCP2* variant was a strong predictor of T2D with earlier onset. The promoter variant in the *UCP2* gene has been associated with increased expression of the gene in adipose tissue [41]. If this variant is associated with increased *UCP2* mRNA levels in human pancreatic β-cells (which is not known), this could result in increased uncoupling and, in turn, in decreased formation of ATP and impaired insulin secretion.

### *KCNJ11*

Genetic variants in the *KCNJ11* gene have not only been associated with T2D [16,18], but also with a severe form of neonatal diabetes [42]. Whereas these neonatal mutations result in a 10-fold activation of the ATP-dependent potassium channel, the E23K variant results in only a 2-fold increase in activity [43]. The *KCNJ11* E23K variant did not significantly increase the risk for T2D in our study's participants. We have no explanation for this finding other than lack of power (the study had only 52% power to detect an effect of *KCNJ11* E23K on risk of developing T2D) or the presence of other unidentified risk factors in the patients with manifest T2D. We did, however, observe an interaction between the EK and/or KK genotypes and impaired β-cell function, supporting a role in insulin secretion.

### Conclusion

In conclusion, we show in a large observational prospective study that genetic variants in candidate genes can predict future T2D, particularly in association with conventional risk factors such as obesity and abnormal glucose tolerance. With accumulating data from prospective studies, it should be possible to define whether there will be a future role for these variants in genetic prediction of T2D and whether these variants will influence response to prevention or treatment.

Although this study is, to our knowledge, the largest of its kind thus far, it still has limited power to detect an effect of low-frequency alleles. It is therefore obvious that larger studies with longer follow-up are needed to replicate the findings. One such resource will be the Malmö Diabetes Prevention cohort, in which 22,000 individuals have been followed for more than 20 y. They are presently being restudied to obtain DNA and information on whether they have developed diabetes or not. While waiting for these results, it will be important to create consortia to merge data from available prospective studies.

## Supporting Information

Table S1 Primers and Probes Used in the Study (92 KB PDF).

Table S2 The Number of Individuals Who Developed T2D Carrying Different Risk Factors (93 KB PDF).

### Accession Numbers

The NCBI Entrez (http://www.ncbi.nlm.nih.gov/gquery/gquery.fcgi) accession numbers for the polymorphisms discussed in this paper are *CAPN10* SNP43 (rs3792267), *CAPN10* SNP44 (rs2975760), *IRS1* G972R (rs1801278), *KCNJ11* E23K (rs5219), *PPARG* P12A (rs1801282), and *UCP2* −866G>A (rs659366).

**Patient Summary**

**Background**

Type 2 diabetes, also known as adult onset or non-insulin-dependent diabetes, is increasing in frequency around the world. Many different factors work together to make someone more likely to develop diabetes, including factors in their environment—for example, the food they eat—and in their family background—the genes they inherited from their parents. Many studies have been done looking at which genes are associated with diabetes, but few have tried to see whether it is possible to predict who will get diabetes in future from looking at a person's genes before any symptoms develop.

**Why Was This Study Done?**

These authors wanted to look at changes in five genes previously shown to be associated with diabetes in a group of people who were to be followed prospectively—that is, from before they developed diabetes—and see if it was possible to predict who would get diabetes.

**What Did the Researchers Do and Find?**

They studied 2,293 people in Finland who were family members or spouses of people with type 2 diabetes, but who themselves did not have diabetes. They followed these people for up to 12 years, starting in 1990. In total, 132 of these individuals (6%) developed diabetes during this time. They found that changes in two of the genes, *PPARG* (which is involved in how the body regulates fat tissue) and *CAPN10* (which is involved in modifying certain proteins), were associated with people having a higher chance of getting type 2 diabetes. This chance was increased substantially when the participants already had slightly raised blood glucose, and a high body mass index.

**What Do These Findings Mean?**

In some people, it does seem possible to use certain genes to predict whether a person will develop type 2 diabetes. However, environmental factors are also very important, and any risk is much increased in people who are already overweight.

**Where Can I Get More Information Online?**

MedlinePlus has many links to pages of information on diabetes:

http://www.nlm.nih.gov/medlineplus/ency/article/001214.htm

The Finnish Diabetes Association has information on diabetes in general and more specifically for Finland:

http://www.diabetes.fi/english/

## Abbreviations

BMI: body mass index
CI: confidence interval
FPG: fasting plasma glucose concentration
HOMA_IR_: homeostasis model assessment index
HR: hazard ratio
OGTT: oral glucose tolerance test
T2D: type 2 diabetes
SNP: single nucleotide polymorphism

## References

[pmed-0020345-b01] Zimmet P, Alberti KG, Shaw J (2001). Global and societal implications of the diabetes epidemic. Nature.

[pmed-0020345-b02] Bonadonna RC (2004). Alterations of glucose metabolism in type 2 diabetes mellitus. An overview. Rev Endocr Metab Disord.

[pmed-0020345-b03] Chan JM, Rimm EB, Colditz GA, Stampfer MJ, Willett WC (1994). Obesity, fat distribution, and weight gain as risk factors for clinical diabetes in men. Diabetes Care.

[pmed-0020345-b04] Altshuler D, Hirschhorn JN, Klannemark M, Lindgren CM, Vohl MC (2000). The common PPARgamma Pro12Ala polymorphism is associated with decreased risk of type 2 diabetes. Nat Genet.

[pmed-0020345-b05] Barroso I, Luan J, Middelberg RP, Harding AH, Franks PW (2003). Candidate gene association study in type 2 diabetes indicates a role for genes involved in β-cell function as well as insulin action. PLoS Biol.

[pmed-0020345-b06] Florez JC, Hirschhorn J, Altshuler D (2003). The inherited basis of diabetes mellitus: Implications for the genetic analysis of complex traits. Annu Rev Genomics Hum Genet.

[pmed-0020345-b07] Laukkanen O, Pihlajamaki J, Lindstrom J, Eriksson J, Valle TT (2004). Common polymorphisms in the genes regulating the early insulin signalling pathway: Effects on weight change and the conversion from impaired glucose tolerance to type 2 diabetes. The Finnish Diabetes Prevention Study. Diabetologia.

[pmed-0020345-b08] Parikh H, Groop L (2004). Candidate genes for type 2 diabetes. Rev Endocr Metab Disord.

[pmed-0020345-b09] Lindi VI, Uusitupa MI, Lindstrom J, Louheranta A, Eriksson JG (2002). Association of the Pro12Ala polymorphism in the PPAR-gamma2 gene with 3-year incidence of type 2 diabetes and body weight change in the Finnish Diabetes Prevention Study. Diabetes.

[pmed-0020345-b10] Memisoglu A, Hu FB, Hankinson SE, Liu S, Meigs JB (2003). Prospective study of the association between the proline to alanine codon 12 polymorphism in the PPARgamma gene and type 2 diabetes. Diabetes Care.

[pmed-0020345-b11] Andrulionyte L, Zacharova J, Chiasson JL, Laakso M (2004). Common polymorphisms of the PPAR-gamma2 (Pro12Ala) and PGC-1alpha (Gly482Ser) genes are associated with the conversion from impaired glucose tolerance to type 2 diabetes in the STOP-NIDDM trial. Diabetologia.

[pmed-0020345-b12] Horikawa Y, Oda N, Cox NJ, Li X, Orho-Melander M (2000). Genetic variation in the gene encoding calpain-10 is associated with type 2 diabetes mellitus. Nat Genet.

[pmed-0020345-b13] Weedon MN, Schwarz PE, Horikawa Y, Iwasaki N, Illig T (2003). Meta-analysis and a large association study confirm a role for calpain-10 variation in type 2 diabetes susceptibility. Am J Hum Genet.

[pmed-0020345-b14] MSong Y, Niu T, Manson JE, Kwiatkowski DJ, Liu S (2004). Are variants in the CAPN10 gene related to risk of type 2 diabetes? A quantitative assessment of population and family-based association studies. Am J Hum Genet.

[pmed-0020345-b15] Orho-Melander M, Klannemark M, Svensson MK, Ridderstrale M, Lindgren CM (2002). Variants in the calpain-10 gene predispose to insulin resistance and elevated free fatty acid levels. Diabetes.

[pmed-0020345-b16] Gloyn AL, Weedon MN, Owen KR, Turner MJ, Knight BA (2003). Large-scale association studies of variants in genes encoding the pancreatic beta-cell KATP channel subunits Kir6.2 (KCNJ11) and SUR1 (ABCC8) confirm that the KCNJ11 E23K variant is associated with type 2 diabetes. Diabetes.

[pmed-0020345-b17] Laukkanen O, Pihlajamaki J, Lindstrom J, Eriksson J, Valle TT (2004). Polymorphisms of the SUR1 (ABCC8) and Kir6.2 (KCNJ11) genes predict the conversion from impaired glucose tolerance to type 2 diabetes. The Finnish Diabetes Prevention Study. J Clin Endocrinol Metab.

[pmed-0020345-b18] Florez JC, Burtt N, De Bakker PI, Almgren P, Tuomi T (2004). Haplotype structure and genotype-phenotype correlations of the sulfonylurea receptor and the islet ATP-sensitive potassium channel gene region. Diabetes.

[pmed-0020345-b19] Marchetti P, Lupi R, Federici M, Marselli L, Masini M (2002). Insulin secretory function is impaired in isolated human islets carrying the Gly(972)→Arg IRS-1 polymorphism. Diabetes.

[pmed-0020345-b20] Jellema A, Zeegers MP, Feskens EJ, Dagnelie PC, Mensink RP (2003). Gly972Arg variant in the insulin receptor substrate-1 gene and association with type 2 diabetes: A meta-analysis of 27 studies. Diabetologia.

[pmed-0020345-b21] Zeggini E, Parkinson J, Halford S, Owen KR, Frayling TM (2004). Association studies of insulin receptor substrate 1 gene (IRS1) variants in type 2 diabetes samples enriched for family history and early age of onset. Diabetes.

[pmed-0020345-b22] Florez JC, Sjogren M, Burtt N, Orho-Melander M, Schayer S (2004). Association testing in 9,000 people fails to confirm the association of the insulin receptor substrate-1 G972R polymorphism with type 2 diabetes. Diabetes.

[pmed-0020345-b23] van Dam RM, Hoebee B, Seidell JC, Schaap MM, Blaak EE (2004). The insulin receptor substrate-1 Gly972Arg polymorphism is not associated with type 2 diabetes mellitus in two population-based studies. Diabet Med.

[pmed-0020345-b24] Wang H, Chu WS, Lu T, Hasstedt SJ, Kern PA (2004). Uncoupling protein-2 polymorphisms in type 2 diabetes, obesity, and insulin secretion. Am J Physiol Endocrinol Metab.

[pmed-0020345-b25] Sasahara M, Nishi M, Kawashima H, Ueda K, Sakagashira S (2004). Uncoupling protein 2 promoter polymorphism −866G/A affects its expression in beta-cells and modulates clinical profiles of Japanese type 2 diabetic patients. Diabetes.

[pmed-0020345-b26] D'Adamo M, Perego L, Cardellini M, Marini MA, Frontoni S (2004). The −866A/A genotype in the promoter of the human uncoupling protein 2 gene is associated with insulin resistance and increased risk of type 2 diabetes. Diabetes.

[pmed-0020345-b27] Bulotta A, Ludovico O, Coco A, Di Paola R, Quattrone A (2005). The common −866G/A polymorphism in the promoter region of the UCP2 gene is associated with reduced risk of type 2 diabetes in Caucasians from Italy. J Clin Endocrinol Metab.

[pmed-0020345-b28] Krempler F, Esterbauer H, Weitgasser R, Ebenbichler C, Patsch JR (2002). A functional polymorphism in the promoter of UCP2 enhances obesity risk but reduces type 2 diabetes risk in obese middle-aged humans. Diabetes.

[pmed-0020345-b29] Chan CB, De Leo D, Joseph JW, McQuaid TS, Ha XF (2001). Increased uncoupling protein-2 levels in beta-cells are associated with impaired glucose-stimulated insulin secretion: Mechanism of action. Diabetes.

[pmed-0020345-b30] Groop L, Forsblom C, Lehtovirta M, Tuomi T, Karanko S (1996). Metabolic consequences of a family history of NIDDM (the Botnia study): Evidence for sex-specific parental effects. Diabetes.

[pmed-0020345-b31] Lyssenko V, Almgren P, Anevski D, Perfekt R, Lahti K (2005). Predictors of and longitudinal changes in insulin sensitivity and secretion preceding onset of type 2 diabetes. Diabetes.

[pmed-0020345-b32] World Health Organization (1999). Definition, diagnosis, and classification of diabetes mellitus and its complications. Report of a WHO consultation. Part 1: Diagnosis and classification of diabetes mellitus.

[pmed-0020345-b33] Levy JC, Matthews DR, Hermans MP (1998). Correct homeostasis model assessment (HOMA) evaluation uses the computer program. Diabetes Care.

[pmed-0020345-b34] Klein JP, Moeschberger ML (2003). Survival analysis: Techniques for censored and truncated data, 2nd ed.

[pmed-0020345-b35] Deeb SS, Fajas L, Nemoto M, Pihlajamaki J, Mykkanen L (1998). A Pro12Ala substitution in PPARgamma2 associated with decreased receptor activity, lower body mass index and improved insulin sensitivity. Nat Genet.

[pmed-0020345-b36] Stumvoll M, Haring H (2002). Reduced lipolysis as possible cause for greater weight gain in subjects with the Pro12Ala polymorphism in PPARgamma2?. Diabetologia.

[pmed-0020345-b37] Nicklas BJ, van Rossum EF, Berman DM, Ryan AS, Dennis KE (2001). Genetic variation in the peroxisome proliferator-activated receptor-gamma2 gene (Pro12Ala) affects metabolic responses to weight loss and subsequent weight regain. Diabetes.

[pmed-0020345-b38] Luan J, Browne PO, Harding AH, Halsall DJ, O'Rahilly S (2001). Evidence for gene-nutrient interaction at the PPARgamma locus. Diabetes.

[pmed-0020345-b39] Baier LJ, Permana PA, Yang X, Pratley RE, Hanson RL (2000). A calpain-10 gene polymorphism is associated with reduced muscle mRNA levels and insulin resistance. J Clin Invest.

[pmed-0020345-b40] O'Rahilly S, Spivey RS, Holman RR, Nugent Z, Clark A (1987). Type II diabetes of early onset: A distinct clinical and genetic syndrome?. Br Med J.

[pmed-0020345-b41] Esterbauer H, Schneitler C, Oberkofler H, Ebenbichler C, Paulweber B (2001). A common polymorphism in the promoter of UCP2 is associated with decreased risk of obesity in middle-aged humans. Nat Genet.

[pmed-0020345-b42] Gloyn AL, Pearson ER, Antcliff JF, Proks P, Bruining GJ (2004). Activating mutations in the gene encoding the ATP-sensitive potassium-channel subunit Kir6.2 and permanent neonatal diabetes. N Engl J Med.

[pmed-0020345-b43] Nichols CG, Koster JC (2002). Diabetes and insulin secretion: Whither KATP?. Am J Physiol Endocrinol Metab.

