# Supplementary material for: Genetic Prediction of Future Type 2 Diabetes
Source: PLoS Med. 2005 Nov 1;2(12):e345. doi: 10.1371/journal.pmed.0020345 (PMC1274281; doi:10.1371/journal.pmed.0020345)
Supplement: Supplementary file 1 [file pmed.0020345.st001.pdf]

SNP	Method		Primers		Detection primers / Probes
			Forward 5'-3'	Reverse 5'-3'	
rs1801282	Agarose gel electrophoresis	PCR-RFLP	GATAGAGACAAA ATATCAGTG	GTATCAGTGAAG GAATCGCTTTCC G	
rs3792267 and rs2975768	ABI-3100	SBE-PCR	GCTGGCTGGTGACAT CAGTGC	TCAGGTTCCATCTTT CTGCCAG	GGCTTAGCCTCACCTTCAAA and GACTGCAGGGCGCTCACGCTTG CTG
rs2975760	ABI-7900	Allelic-discrimination	GCAGGGCGCTCACG	CCTCACCAAGTCAA GGCTTAGC	VIC-CCTTACTTCGCAGCAAG FAM-CCTTACTTCACAGCAAG
rs659366	ABI-7900	Allelic-discrimination	GGGCCTGGTTCGCCT TTAATT	GCCAGAGGGCCCAA TTGTT	VIC-CACGCGTCAGTTAC FAM-TTCACGCATCAGTTAC
rs1801278	ABI-7900	Allelic-discrimination	GGGTAGGCCTGCAA ATGCTA	GGTCGAGATGGGCA GACT	VIC-CAGCCCCGGGAGGT FAM-CAGCCCTGGGAGGT
rs5219	ABI-7900	Allelic-discrimination	CAGTTGCCTTTCTTG GACACAAA	CCGAGGAATACGTG CTGACA	VIC-CCTGCCAAGCCCAG FAM-CTGCCGAGCCCAG
