# Supplementary material for: Genetic Prediction of Future Type 2 Diabetes
Source: PLoS Med. 2005 Nov 1;2(12):e345. doi: 10.1371/journal.pmed.0020345 (PMC1274281; doi:10.1371/journal.pmed.0020345)
Supplement: Supplementary file 2 [file pmed.0020345.st002.pdf]

Polymorphism		FPG<5.6 and BMI<30		FPG ≥5.6 and BMI<30		FPG<5.6 and BMI≥30		FPG≥5.6 and BMI≥30	
		Converters	Non-converters	Converters	Non-converters	Converters	Non-converters	Converters	Non-converters
PPARG P12A	PP	28	786	47	613	7	63	27	91
	PA/AA	4	263	12	231	4	44	3	39
CAPN10 SNP43 G>A	GG	15	518	30	409	4	55	21	63
	GA/AA	15	508	28	415	7	50	9	62
CAPN10 SNP44 T>C	TT	19	640	37	527	9	57	26	77
	TC/CC	13	397	20	316	2	48	4	52
CAPN10 SNP43/44	GG/TT	6	252	15	188	3	20	18	31
	Other	24	765	41	630	8	83	12	93
UCP2 –866G>A	GG	12	363	30	318	6	43	10	58
	GA/AA	19	660	29	504	5	60	20	68
IRS1 G972R	GG	26	874	49	684	11	88	22	105
	GR/RR	6	130	10	123	0	15	7	18
KCNJ11 E23K	EE	11	252	13	189	4	23	6	31
	EK/KK	19	781	46	637	7	85	24	98
PPARG/CAPN10 -44	PP/TT	16	481	30	380	5	36	23	53
	Other	16	549	27	455	6	68	7	76
PPARG/CAPN10 -43/44	PP/GG/TT	5	195	15	140	3	17	17	21
	Other	25	815	41	673	8	85	13	103
